# Vortex dynamics and frequency splitting in vertically coupled nanomagnets

**DOI:** 10.1038/s41598-017-01222-4

**Published:** 2017-04-25

**Authors:** M. E. Stebliy, S. Jain, A. G. Kolesnikov, A. V. Ognev, A. S. Samardak, A. V. Davydenko, E. V. Sukovatitcina, L. A. Chebotkevich, J. Ding, J. Pearson, V. Khovaylo, V. Novosad

**Affiliations:** 1 School of Natural Sciences, Far Eastern Federal University, Vladivostok, 690091 Russia; 2 Argonne National Laboratory, Materials Science Division, Argonne, 60439 Ilinois United States; 3 National University of Science and Technology (“MISiS”), Moscow, 119049 Russia; 4 National Research South Ural State University, Chelyabinsk, 454080 Russia; 5 Western Digital, 1710 Automation Pkwy, San Jose, 95131 California United States

## Abstract

We explored the dynamic response of a vortex core in a circular nanomagnet by manipulating its dipole-dipole interaction with another vortex core confined locally on top of the nanomagnet. A clear frequency splitting is observed corresponding to the gyrofrequencies of the two vortex cores. The peak positions of the two resonance frequencies can be engineered by controlling the magnitude and direction of the external magnetic field. Both experimental and micromagnetic simulations show that the frequency spectra for the combined system is significantly dependent on the chirality of the circular nanomagnet and is asymmetric with respect to the external bias field. We attribute this result to the strong dynamic dipole-dipole interaction between the two vortex cores, which varies with the distance between them. The possibility of having multiple states in a single nanomagnet with vertical coupling could be of interest for magnetoresistive memories.

## Introduction

A magnetic vortex (MV) is characterized by an in-plane rotation of magnetization and an out-of-plane magnetic component in the center^1^ of the magnet. MV is a stable state with linear response to a magnetic field^2^ and to spin-polarized current^3^ in a wide range of excitation amplitudes. The gyrotropic mode of a vortex core attracts a lot of attention, potentially due to the complex dependence of its frequency to an external field and current, both static and alternating, which can lead to its core reversal^4, 5^. The rich frequency spectrum of vortices make them especially interesting for applications in non-volatile magnetic memory cells^6^, high-frequency generators of spin current^7^, microwave nano-oscillators^8^, carriers for biomedical applications^9^ and bio-functionalized systems for targeted cancer-cell destruction^10^. So far, magnetostatic interactions between vortex cores has been studied extensively in coupled nanomagnets, placed laterally to one another^11^, or in elliptical nanomagnets^12^, where occurrence of multiple vortices is possible. However, vertical coupling between the vortex cores has been studied to a lesser extent^13–15^, partially due to challenges in fabrication, as well as in obtaining desired magnetic configurations in individual nanomagnets. For instance, by using a micromagnetic approach it was shown that the vertical structure consisting of two asymmetrically stacked nanodisks has the following features^16–18^: (i) both disks can contain either a stable vortex or single domain state (SDS); (ii) transition between two states can be induced by an external high frequency magnetic field excitation; (iii) micromagnetic configuration – MV or SDS - can be reliably determined by electrical resistance measurements.

## Results and Discussion

In this work, we investigate a model system of two nanomagnets placed on top of each other, with different geometrical parameters and each having a vortex core with a large contrast in their eigenfrequencies. The vertical dipole-dipole interaction was engineered by controlling the direction and magnitude of a static external field with respect to the two nanomagnets. Distinct frequency splitting was observed in the equilibrium state corresponding to the eigenfrequencies of the two vortex cores. The difference in two resonance frequencies was varied depending on the position of two vortices. When the cores are in close proximity to each other, the exchange interaction couples the vortices strongly and reduces the contrast between their excitation frequencies. This contrast rises as the distance between the vortices increases. The vertical configuration of the nanomagnets makes them particularly sensitive to the direction of an external magnetic field, which defines positions of the vortex cores.

**Figure 1:**
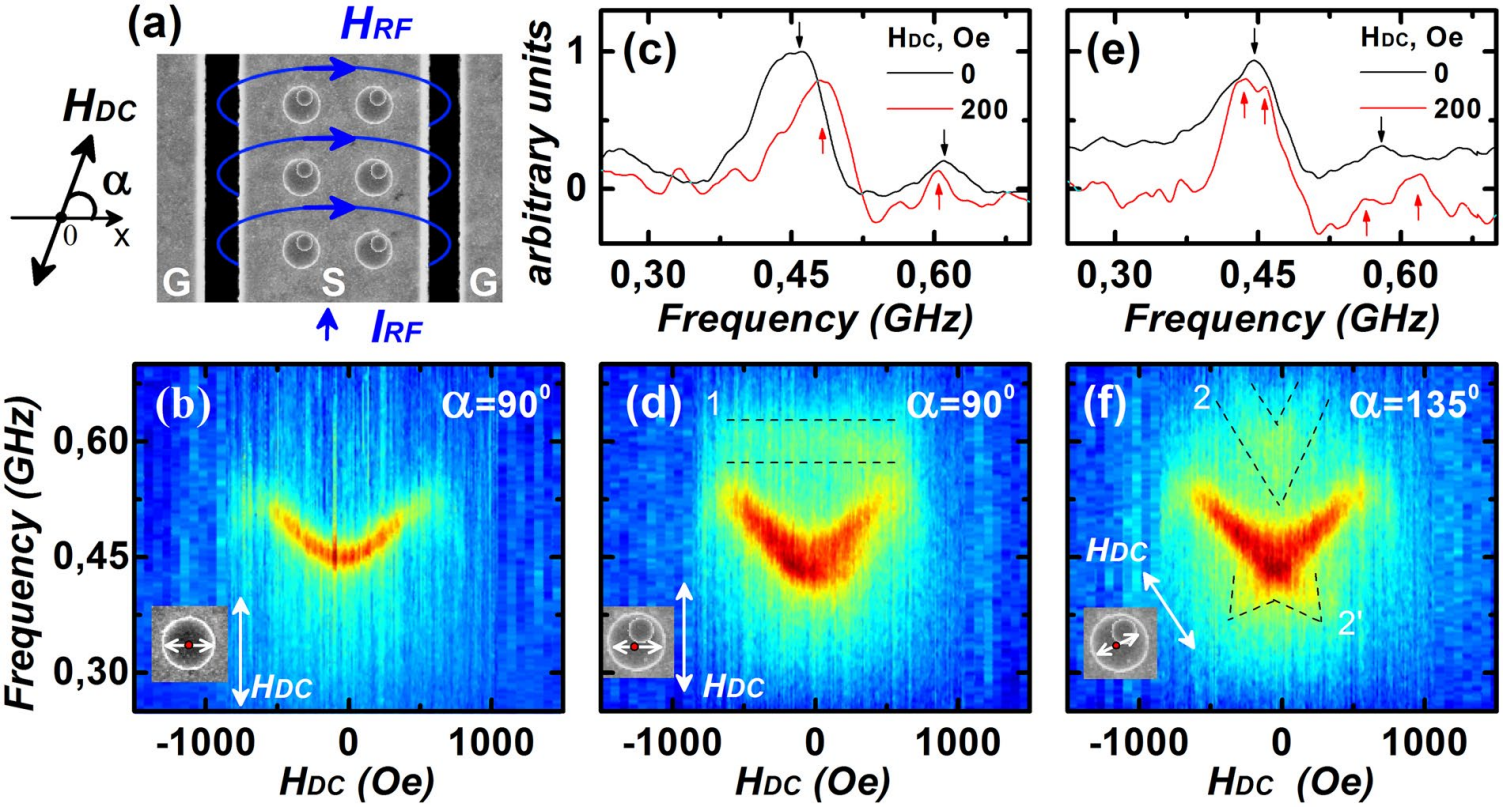
(**a**) SEM image of the vertically coupled nanomagnets placed on the CPW’s signal line. Experimentally measured absorption spectra as a function of external bias field for single big disks are shown in (**b**) and for the coupled system of two disks at α = 90° and 135° are shown in (**d**) and (**f**), respectively. (**c**) and (**d**) Plots compare the dynamic response obtained in the remanent state (black lines) and in the applied field of 200 Oe (red lines) for these two distinct angles.

The vertically stacked nanomagnets consisting of the small disk (SD) and big disk (BD) with diameters of 200 nm and 600 nm, respectively, was placed on coplanar waveguides (CPW). The distance between the disk centers was ~170 nm and the angle between the horizontal axis and the axis connecting both disk centers was ~70°, Fig. 1(a). Absorption spectra were obtained in the reflection mode using a vector network analyzer as an alternating current source. The excitation frequency was varied from 50 MHz up to 1 GHz. The transmission of alternating current I_RF_ induced an alternating magnetic field H_RF_ and was perpendicular to the CPW. In addition to H_RF_ a constant magnetic field H_DC_ oriented at an angle α relative to the field H_RF_ was applied. The strength of H_DC_ was varied from −1.5 to +1.5 kOe, respectively. The scanning electron microscopy (SEM) image of the corresponding device is shown in Fig. 1(a).

Figure 1(b) shows the absorption spectra for single reference disks with diameter of 600 nm when H_DC_ is perpendicular to H_RF_. The resonance frequency increases with increase in the external field due to vortex confinement as it approaches the edges^17^. The width of the resonance absorption line remains almost unchanged and equal to *δf* = 25 MHz, indicating the negligible magnetostatic interaction between two nanomagnets^19^. Figure 1(d) shows the absorption spectra for the vertically coupled system for α = 90°. The white arrow in the inset indicates the direction where the vortex core of BD will move under the influence of an external magnetic field. One of the differences easily observed in this spectra is the presence of a constant absorption at ~600 MHz. The linewidth of the fundamental resonance frequency also increases to *δf* ≈ 70 MHz, respectively. The observed features change drastically when α is increased to 135°, as shown in Fig. 1(e). Depending on the direction of the external magnetic field, the vortex core of BD will be closest to the vortex core of SD, or it will be farthest. This should make the frequency response of the system asymmetric with respect to the external magnetic field. The constant absorption peak at ~600 MHz (in Fig. 1(d)) now becomes field dependent (Fig. 1(f)), and there is an additional frequency response observed at H_DC_ ~ 60 Oe. The difference between these two scenarios can be further observed in Fig. 1(c) and (e) comparing the spectra obtained in remanence and in the magnetic field of 200 Oe applied at the angle α = 90° and 135°, respectively.

In order to understand this complex frequency response from the system of vertically coupled disks, micromagnetic simulations were performed using OOMMF^20^. The calculations were done in two stages using methods described in ref. 21. In line with the previous work^22, 23^, absorption spectra were computed for both zero field and with varying static external field in the range of ±1.5 kOe. The dynamic characteristics of the combined system were studied with two approaches, (see details in Supplementary info): (i) computing the dynamical susceptibility spectra using the Fourier transformation from time to frequency domain and (ii) construction of a spatial distribution of the imaginary part of susceptibility as a function of the frequency and the phase-shift between the exciting magnetic field *H*_*RF*_ and the local magnetization. Thus, the first approach was used to plot spectral dependences of the imaginary part of magnetic susceptibility of the coupled structures, and the second one was employed to track the position and areas involved in the energy absorption at the resonance frequencies.

**Figure 2:**
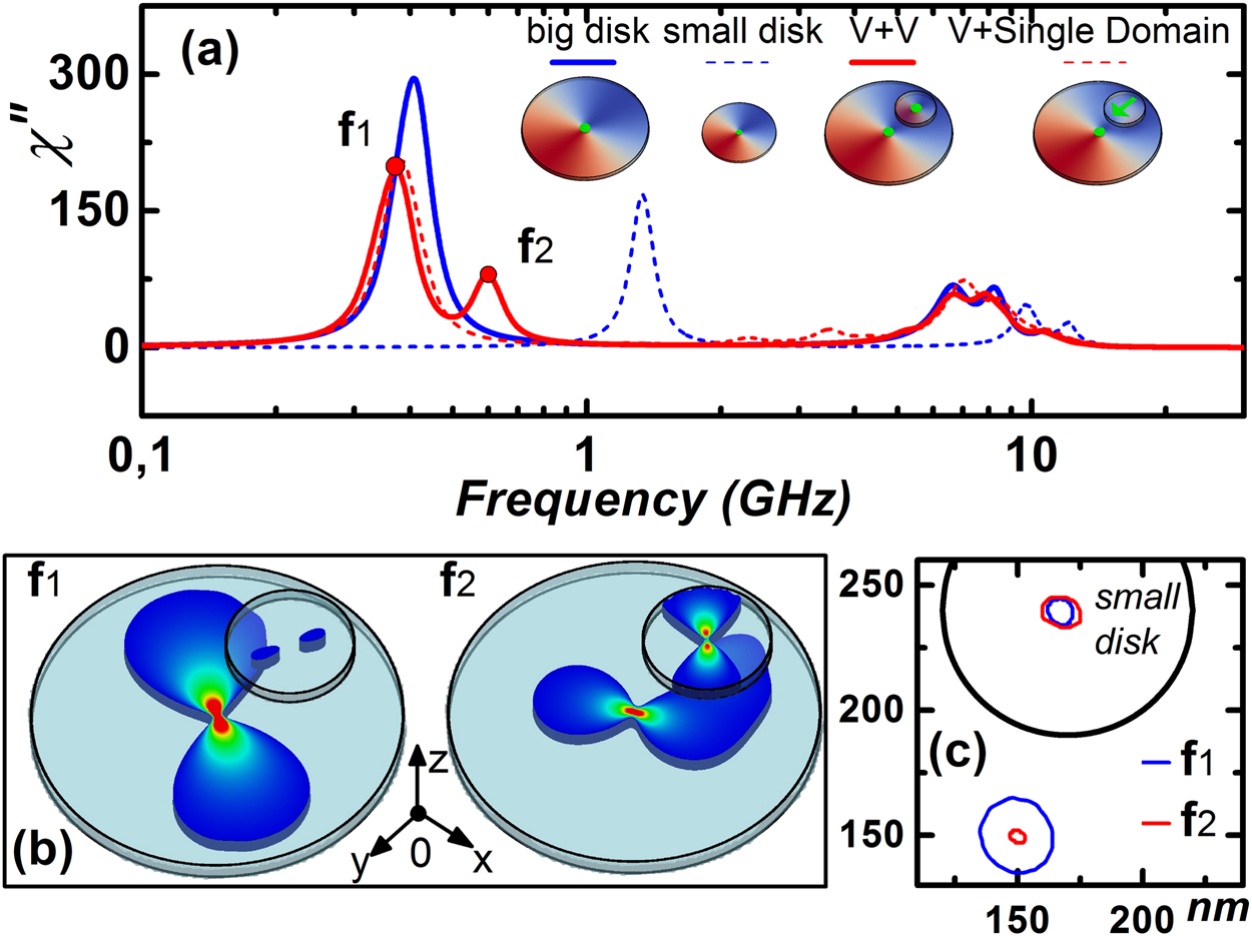
(**a**) Simulated spectral dependencies of the imaginary part of magnetic susceptibility for H_DC_ = 0 for the vertically coupled system with V + V and V+ Single Domain states. (**b**) Spatial distribution of magnetic susceptibility for the coupled system at resonance peaks of f1 and f2. (**c**) Zoomed in part of the structure, where the motion of the vortex cores under the presence of H_RF_ is localized.

We attempted to analyze the experimental data based on simulation results obtained using the two methods described above. In the case of a single disk with vortex configuration, the spectral dependence of magnetic susceptibility has a pronounced resonance peak corresponding to the gyrotropic mode of vortex core motion^24, 25^. In the case of a small disk, this resonance peak shifts to higher frequencies. To assess the magnetic susceptibility of the coupled structure, spectra of the imaginary part for the big and small disks were plotted, as shown in Fig. 2(a). In the absence of the bias field, the resonance frequencies for BD and SD are 0.4 GHz and 1.3 GHz, respectively. The obtained spectrum contains double peaks in the high frequency region corresponding to azimuthal spin waves with index n = 0 and m = ±1 ref. 4. The discussion of high frequency resonance peaks is out of the scope of this paper. For completeness, two cases of magnetic configurations in the small disk were considered: single-domain and vortex^16^. Calculations show that in the first case, the spectral dependence for the coupled structure is not qualitatively different from the response of a single nanomagnet. However, if SD has a vortex state, the spectrum qualitatively changes, and there is an additional resonance peak observed near the main peak (f2).

To understand the origins of two resonance peaks, a spatial distribution of magnetic susceptibility at those frequencies was plotted in Fig. 2(b). At f1 (0.38 GHz), the contribution of the small disk to the system absorption is minimal and equals to 0.7%. The main absorption area is centered around the vortex core of BD and has a dumbbell profile. At f2 (0.59 GHz), the absorption contribution of SD is significantly increased up to 24%. Due to the magnetization perturbations in SD, the magnetostatic interaction between the disks increases and the area beneath SD contributes to the absorption process. At f1, the absorbance of this area was 5%, but at f2 this contribution increases up to 23%. In addition, vortex core trajectories and velocities were also estimated at two resonance frequencies. The vortex core in BD is characterized by a circular orbit movement around the equilibrium position. When the resonance frequency shifts from f1 to f2, the trajectory diameter decreases sharply from 60 nm to 12 nm, and the tangential velocity component drops from 55 down to 47 m/s. Opposite behavior is observed in SD: transition to the higher frequency increases the trajectory diameter from 20 to 30 nm and the core velocity from 25 to 48 m/s. Thus, it can be concluded that the second peak (f2) corresponds to the resonance frequency of SD as a part of the coupled system.

**Figure 3:**
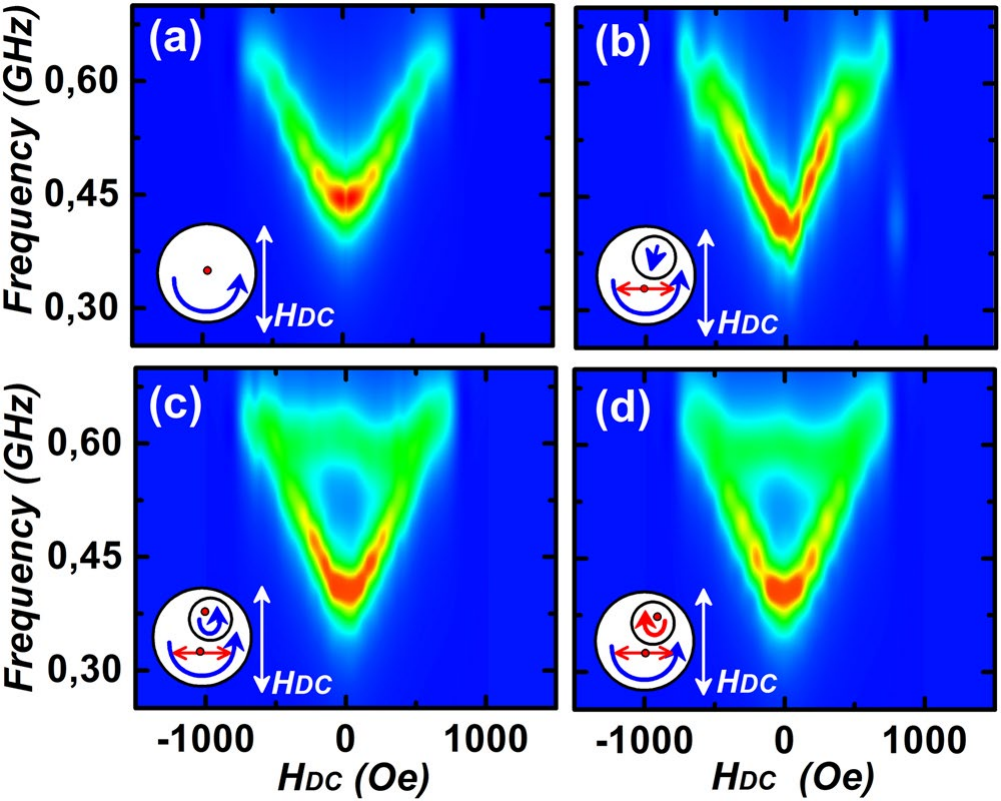
Simulated absorption spectrum as a function of H_DC_ for a single BD is shown in (**a**) and for a coupled system with single-domain configuration of the SD is shown in (**b**). Spectra in (**c**) and (**d**) are for the cases with both SD and BD in vortex configuration, but SD with two different chiralities. Fields H_RF_ and H_DC_ are orthogonal to each other.

Absorption spectra in the presence of external bias fields were also calculated and summarized as shown in Fig. 3 for α = 90°. There is a qualitative agreement between the simulated response from a single nanomagnet (Fig. 3(a)) with the experimental data shown in Fig. 1(b). However, the spectra obtained when SD is in single-domain state, does not agree with the experimental observation for the coupled system shown in Fig. 1(c). On the contrary, when SD is in the vortex state, it is in agreement with the experimental response owing to the presence of f2 near ~600 MHz. Figure 3(c) and (d) show that the coupled system is insensitive to the chirality of SD and the responses are symmetric around zero field.

**Figure 4:**
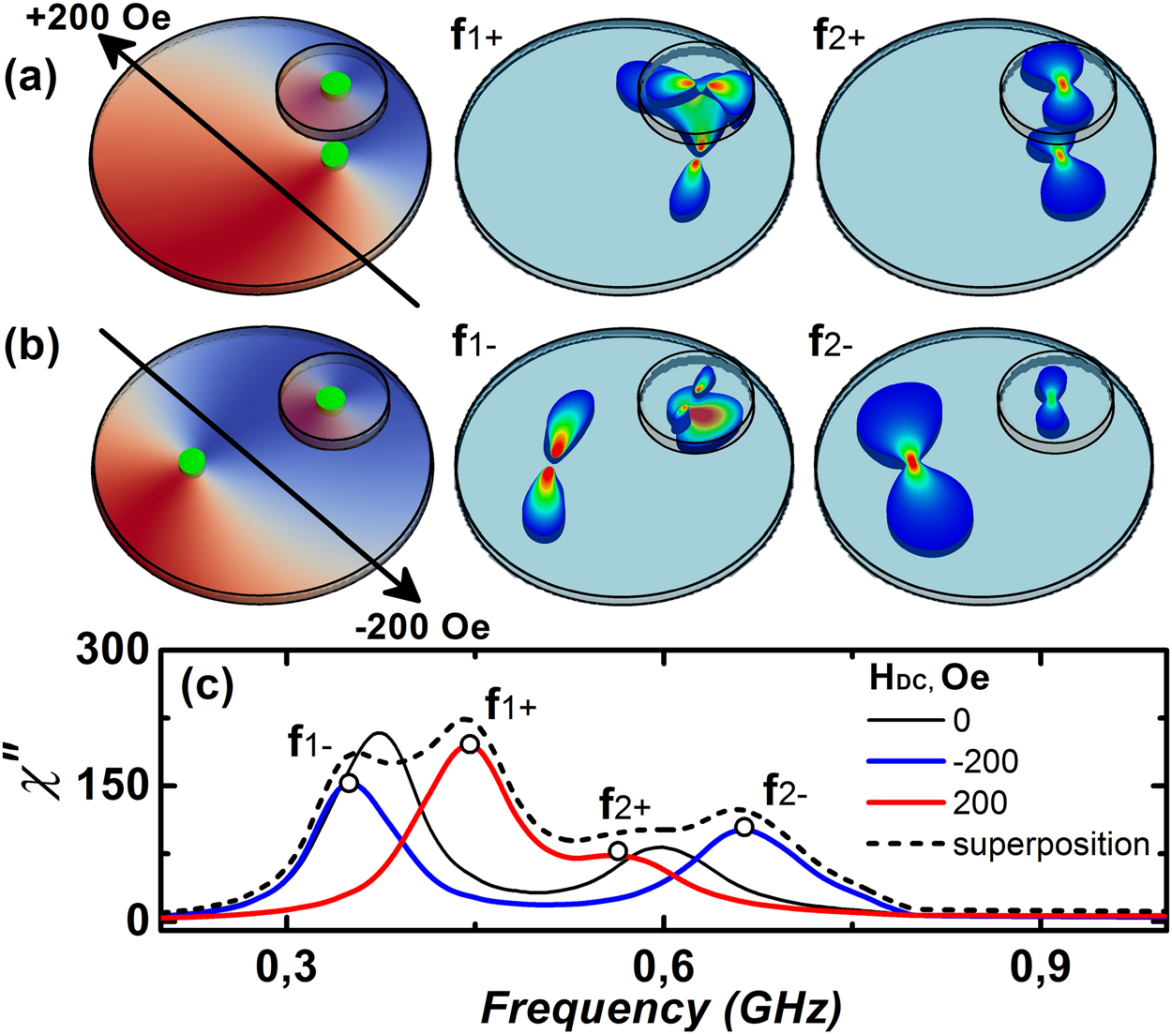
Simulation results of initial magnetization configurations and corresponding spatial distributions of magnetic susceptibility for H_DC_ = +200 Oe (**a**) and −200 Oe (**b**). The respective resonance peaks are marked as f1+, f2+, f1−, f2−. Spectral dependences of χ^″^ for three cases: H_DC_ = 0 Oe (black line), +200 Oe (blue line), −200 Oe (red line) at α = 135° and their superposition (dashed line) are shown in (**c**).

Excitation spectra become interesting when α is increased to 135°. The frequency response of the coupled system is now very much dependent on the direction of the external magnetic field. Two vortex cores are in close proximity for H_DC_ = +200 Oe (as shown in Fig. 4(a)), and they are farthest for H_DC_ = −200 Oe, as shown in Fig. 4(b). The absorption spectra of the magnetic susceptibility for the equilibrium state, as well as for H_DC_ = ±200 Oe is shown in Fig. 4(c). For H_DC_ = 0, the low frequency resonance peak corresponds to the gyrotropic motion of the vortex core in BD (f1), and the low amplitude resonance peak at higher frequency corresponds to the vortex motion in SD (f2). It should be noted that the area of BD located underneath SD influences the excitation spectrum for both the vortex cores, and, therefore, its contribution cannot be uniquely tied to any one of the resonance frequencies.

For H_DC_ = +200 Oe, two vortex cores are strongly coupled to each other and, therefore, their magnetostatic interaction generates a shift in resonance frequencies, bringing f1 and f2 closer to one another as shown in Fig. 4(c). The coupling strength reduces as two cores move away from each other, as in the case of H_DC_ = −200 Oe, thus, increasing the contrast between f1 and f2, beyond their equilibrium resonance frequencies. It is known that the dipole-dipole interaction affects the gyrotropic motion of vortex cores and results in frequency splitting^19, 26–28^ in laterally coupled devices. However, in this work, similar interaction has been shown to exhibit a frequency splitting in vertically coupled nanomagnets as well, which has even higher potential for storage devices.

**Figure 5:**
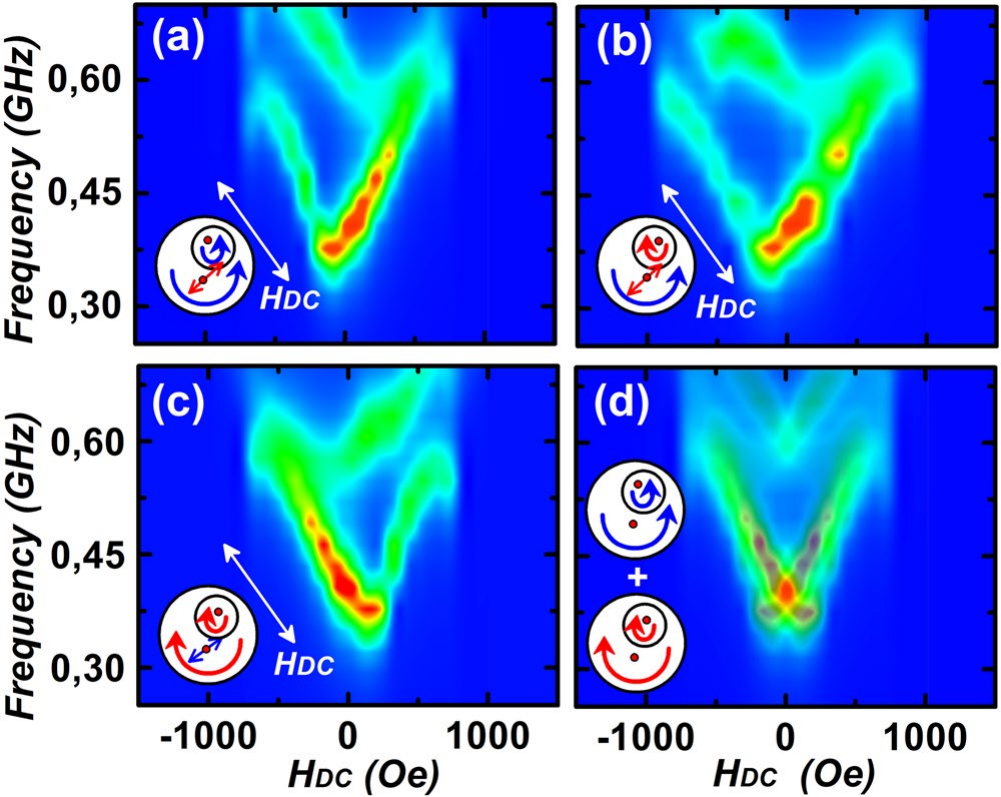
Simulated absorption spectra of the coupled system for α = 135° are shown. In (**a**) and (**c**) are for cases of two vortices with the same chiralities. The spectra in (**b**) correspond to vortices with opposite chiralities. Spectra in (**d**) correspond to the case of co-existence of disks with different chirality combinations in the system and they were obtained as a result of superposition of data shown in (**a**) and (**c**).

Absorption spectra for the entire range of H_DC_ for a particular chirality combination are shown in Fig. 5(a). Asymmetry of the spectra near zero field is clearly evident. Choosing a different chirality for SD does not alter the response for the coupled system, as shown in Fig. 5(b). However, chirality of BD changes the axis of symmetry of the absorption spectra, as seen in Fig. 5(c). Thus, assuming that experimentally, both the chirality combinations of BD are present, one can superimpose the response of Fig. 5(b) and (c) to obtain Fig. 5(d). The experimental spectra obtained in Fig. 1(d) are in good agreement with the simulated response of Fig. 5(d) and strongly indicates that the complex frequency spectra seen is due to variation in the dynamic coupling strength of two vortex cores, which results in three distinct resonance peaks at any finite value of the external magnetic field.

## Summary

In summary, we have demonstrated the successful fabrication of vertically coupled nanomagnets, whose interaction strength dominates the excitation frequencies of two vortex cores present in the nanomagnets. By engineering the direction and magnitude of an external biasing field, specific contrast between the excitation frequencies can be obtained. Due to the asymmetric positioning of the small disk on top of the big disk, asymmetry in the resonance spectra is also observed. Evidently, by manipulating the location of the small disk on top the big one, further engineering of the frequency spectrum can be implemented. The possibility of having multiple states in a single nanomagnet with vertical coupling could be of interest for magnetoresistive memories.

## Methods

### Sample preparation

The system of magnetostatically coupled nanodisks was lithographically patterned on coplanar waveguides (CPW) made of Au (300 nm)/Cr (3 nm) with the 4 μm wide and 1.5 mm long signal line. Each structure consisted of two Ni_80_Fe_20_ nanodisks of thickness t = 35 nm and diameters D = 600 nm and d = 200 nm for the big and small disks, correspondingly. The small disk (SD) was placed on the top of the big disk (BD) and was separated by 3 nm thick Cu layer to avoid direct and indirect ferromagnetic exchange. Detailed information of the fabrication process can be found elsewhere^18^. SD was shifted relative to the center of BD by 170 nm, so that the angle between the CPW’s signal line and the axis connecting centers of disks was 70°. Single layer Py nanodisks with D = 600 nm and t = 35 nm were also fabricated as a reference. The distance between each structure was 1 μm in order to avoid the magnetostatic interaction between them.

### Simulation parameters

For simulations, standard parameters for Py were utilized: saturation magnetization M_s_ = 800 erg/cm^3^, exchange constant A = 13 × 10^−7^ erg/m, Gilbert damping α = 0.05. The two disk system was divided into cells of the size 5 × 5 × 35 nm^3^. To ensure the accuracy of micromagnetic simulations, the size of the elemental cells was kept smaller than the characteristic exchange length of Py. The exchange coupling between nanodisks was excluded in order to imitate the presence of a non-magnetic interlayer.

## Electronic supplementary material

Supplementary info
