# Supplementary material for: Vortex dynamics and frequency splitting in vertically coupled nanomagnets
Source: Sci Rep. 2017 Apr 25;7:1127. doi: 10.1038/s41598-017-01222-4 (PMC5430672; doi:10.1038/s41598-017-01222-4)
Supplement: Supplementary file 1 — Supplementary info [file 41598_2017_1222_MOESM1_ESM.doc]

**Vortex dynamics and frequency splitting in vertically coupled nanomagnets**

M. E. Stebliy^1^, S. Jain^2, *)^ , A. G. Kolesnikov^1^, A.V. Ognev^1^, A.S. Samardak^1, #)^, A.V. Davidenko^1^, E.V. Sukovatitcina^1^, L.A. Chebotkevich^1^, J. Ding^2^, J. Pearson^2^, V. Khovaylo^3,4^, and V. Novosad^2, #)^

^1^School of Natural Sciences, Far Eastern Federal University, Vladivostok, Russia

^2^Argonne National Laboratory, Materials Science Division, Argonne, IL 60439, United States

^3^ National University of Science and Technology (“MISiS”), Moscow, Russia

^4^ National Research South Ural State University, Chelyabinsk 454080, Russia

**Supplementary Information**

The calculations were done in two stages using methods described in Ref.1. As distinct from the earlier presented results^2,3^, for plotting of absorbtion diagrams we computed the absorption spectra not only in zero external magnetic field, but also in H_DC_ varying in the range ±1.5 kOe. For that at the first stage we defined a stable magnetization state in the vertically stacked nanodisk system in the magnetic field H_DC_, which orientation and value were corresponded to the experiment. At the second stage we determined the dynamical characteristics of the nanostructures with help of two approaches: (i) plotting of dynamical susceptibility spectra using the Fourier transformation from time to frequency domain and (ii) construction of a spatial distribution of the imaginary part of susceptibility in dependence on the frequency with definition of the phase-shift between the exciting magnetic field *H_RF_* and the local magnetization, Fig. 1(a).

1. The external magnetic field changes in accordance to the law
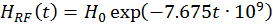
, where t is time in s and
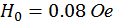
, was applied to the stabilized nanodisk system, Fig. 1(b). The orientation of H_RF_ was fixed for all of the calculated cases and corresponded to the experimental geometry. The co-directional with the field component of magnetization of the system M*_RF_* was registered in the time range from 0 to 30 ns with the step of 1 ps. After the Fourier transformation of the time dependence of the field and magnetization response, we defined the frequency dependences
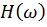
 and
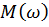
, which were used to compute the spectral dependence of magnetic susceptibility
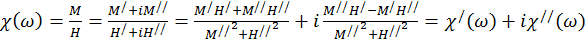
 [19]. The first item is a reactive component of susceptibility describing dynamical response of the system on the change of an external field, Fig. 1(c). The second item is a dissipative component responsible for the energy dissipation in the system, Fig. 1(d). To compare with experimental results on the registration of the system energy absorption, we estimated the imaginary part of susceptibility
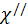
. The spectrum of this component, as a rule, has resonance peaks. To describe the spatial magnetization distribution at peak values, we used the following method.

**
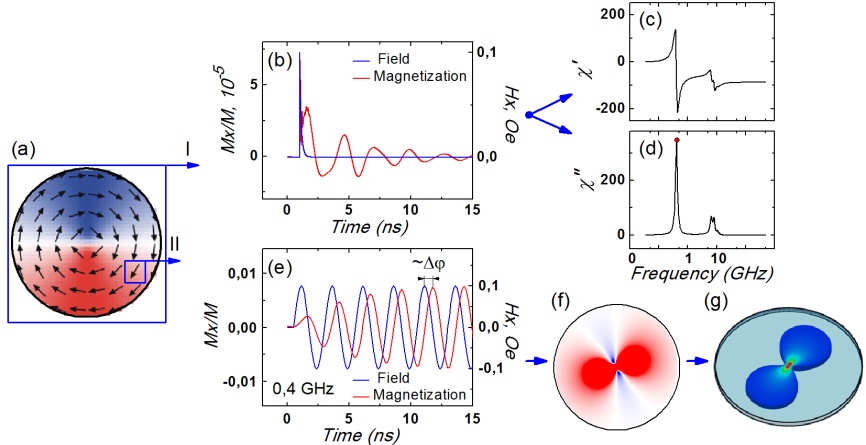
**

Fig.1. (a) Illustration of the two methods of calculation of magnetic susceptibility by the example of a nanodisk with magnetic vortex state. (b) Dependence of the resulting magnetization of the nanodisk under influence of the pulsed magnetic field. Spectral dependences of the real (c) and imaginary (d) parts of magnetic susceptibility. (e) Dependence of magnetization in the selected cell in (a) under action of alternating magnetic field. (f) Spatial distribution of
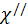
 after isolation of the isosurface (g).

1. An applied to the system sinusoidal alternating magnetic field
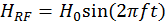
, where f is the resonance frequency and H_0_ = 0.1 Oe, was chosen so that the magnetization change could be specified by the harmonic law. In this case the behavior of magnetization, which can be described as
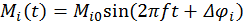
, where Δφ is a phase difference between the external excitation and magnetic response, was recorded in each cell of the system, Fig.1(e). Knowing the amplitudes of the exciting field and of the magnetic response as well as Δφ, it is possible to calculate a magnetic susceptibility distribution: the real part
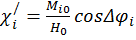
; the imaginary part
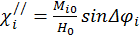
 ^4^. After we made these calculations in each cell, the spatial distributions of the imaginary part for the resonance frequency were plotted, Fig. 1(f). For the visual comparison of areas giving the maximum contribution to the structure absorbance at different frequencies, it is convenient to select the isosurfaces of magnetic susceptibility as shown in Fig. 1(g).

Thus, the first method was used for plotting the spectral dependences of the imaginary part of magnetic susceptibility for vertically couples nanodisk structures, and the second one was employed to reveal the position and size of areas involved in the energy absorption at the resonance frequencies.

**References**

1. Kaya, A., Bain J.A. High frequency susceptibility of closure domain structures calculated using micromagnetic modeling. J. Appl. Phys. 99, 08B708 (2006).

2. Zhang, B., Wang, W., Wang, J. Calculations of three-dimensional magnetic excitations in permalloy nanostructures with vortex state. JMMM 322, 2480 (2010).

3. Vukadinovic, N. High-frequency response of nanostructured magnetic materials. JMMM 321, 2074 (2009).

4. Gerardin, O., Gall, H., Vukadinovic, N. Micromagnetic calculation of the high frequency dynamics of nano-size rectangular ferromagnetic stripes, J. Appl. Phys. 89, 7012 (2001).
